# New Strategies for the Simple and Sensitive Voltammetric Direct Quantification of Se(IV) in Environmental Waters Employing Bismuth Film Modified Glassy Carbon Electrode and Amberlite Resin

**DOI:** 10.3390/molecules26144130

**Published:** 2021-07-07

**Authors:** Małgorzata Grabarczyk, Marzena Adamczyk

**Affiliations:** Department of Analytical Chemistry, Institute of Chemical Sciences, Faculty of Chemistry, Maria Curie-Sklodowska University, 20-031 Lublin, Poland; mgrabarc@poczta.umcs.lublin.pl

**Keywords:** electrochemical measurement, stripping voltammetry, eco-friendly detection of selenium, environmental water sample, resin

## Abstract

An analytical procedure regarding the determination of selenium(IV) by anodic stripping voltammetry exploiting the in situ plated bismuth film electrode is described. Since organics are commonly present in untreated natural water samples, the use of Amberlite XAD-7 resin turns out to be quite important to avoid problems such as the adsorption of these compounds on the working electrode. The optimum circumstances for the detection of selenium in water using differential pulse voltammetry techniques were found to be as follows: 0.1 mol L^−1^ acetic acid, 1.9 × 10^−5^ mol L^−1^ Bi(III), 0.1 g Amberlite XAD-7 resin, and successive potentials of −1.6 V for 5 s and −0.4 V for 60 s, during which the in situ formation of the bismuth film on glassy carbon and the accumulation of selenium took place. The current of the anodic peak varies linearly with the selenium concentration ranging from 3 × 10^−9^ mol L^−1^ to 3 × 10^−6^ mol L^−1^ (r = 0.9995), with a detection limit of 8 × 10^−10^ mol L^−1^. The proposed procedure was used for Se(IV) determination in certified reference materials and natural water samples, and acceptable results and recoveries were obtained.

## 1. Introduction

Research has been focused on the speciation of selenium in the natural environment, as the bioavailability and toxicity of this element depend on its concentration in the environment and consequently in a variety of organisms. Selenium plays a key role in a variety of biochemical and physiological functions in living organisms, but it will be toxic if it is present excessively. Moreover, the nutritional range between essentiality, deficiency, and toxicity in comparison with other microelements is fairly narrow [1]. In addition to the range of concentration, the beneficial and toxic effects of selenium are also dependent on which chemical form it takes. In general, inorganic selenium compounds are more toxic than organic ones [2]. Among the selenium inorganic species, selenite Se(IV) and selenate Se(VI) are the most common water soluble species found in aerobic water sources [1]. Selenite is more bioavailable and approximately 10-fold more toxic than selenate [3]. Therefore, there is a strong need for methods of selenium speciation analysis in order to evaluate its actual risk for the environment in connection with its biological availability and toxicity.

Electroanalytical techniques are undoubtedly a notable option for the trace determination of different metal ions such as selenium(IV), especially in water samples, due to the relative low expense of instrumentation and its operation simplicity. Apart from that, some of these techniques, particularly stripping voltammetry, additionally provide high sensitivity and selectivity as well as in most cases, there is no need to use preliminary sample preparation procedures prior to analysis. Therefore, electrochemical methods are suitable tools for the detection of trace selenium. The stripping voltammetry method makes possible the direct determination of Se(IV), while Se(VI), as electrochemically inactive, is usually reduced to Se(IV) before analysis. Among voltammetric techniques, cathodic stripping voltammetry (CSV) [4,5,6,7,8,9,10,11,12,13,14,15,16,17,18,19,20,21,22] and anodic stripping voltammetry (ASV) [23,24,25,26,27,28,29,30,31,32,33,34,35,36,37,38] have commonly been used for selenium determination. According to our knowledge, currently, there are only few papers with regard to selenium detection procedures by adsorptive stripping voltammetry (AdSV) [39,40,41,42,43,44] available in the literature.

In electrochemical approaches, the analytical performance of a method is strongly dependent on the properties of the materials used in electrode fabrication. An ideal material for electrodes should provide as low a detection limit as possible, the widest range of available potential, and also reproducible response. In addition, the working electrode should also be simple to prepare and friendly to the laboratory environment. As is common knowledge, mercury is the best material to use as a working electrode for voltammetric procedures. Therefore, mercury electrodes were also commonly used in voltammetric procedures for selenium determination, but due to the toxicity of mercury, over the years, electrodes made of mercury have been gradually replaced by other more environmentally friendly electrodes. Mercury electrodes, such as the hanging mercury drop electrode [4,5,6,7,9,10,11,12,13,14,17,18,22,23], mercury film electrode [8], hanging copper amalgam drop electrode [15], and cyclic renewable mercury film silver-based electrode [16], were widely used for the determination of selenium by cathodic stripping voltammetry. The use of solid electrodes made of Ag [19,20] and Cu [21] was also studied. For the determination of Se(IV) by ASV, the gold macroelectrodes [24,25,26,27,28,29,30], gold microelectrodes [30,31,32], or electrodes modified with gold nanoparticles [33,34,35] as well as electrodes modified with O-Phenylenediamine–Nafion [36] were most frequently used. Screen-printed graphite electrode [37] and glassy carbon electrode (GCE) modified with reduced graphene oxide [38] were also used as a working electrode in the determination of selenium by the ASV method. In case of selenium detection by the AdSV method, solid electrodes modified by bismuth film [39,40] as well as mercury electrodes, such as hanging mercury drop electrode [41], mercury film electrode [42], Bi/Hg film-coated electrode [43], and even ceramic composite electrode modified with 2,3-diaminonaphthalene [44] were utilized. In response to the above-mentioned demand, in this work, we have described a new sensitive and selective method for the determination of Se(IV) by ASV using the in situ plated bismuth film electrode as a thin film sensor. The bismuth film is a suitable material to replace mercury electrodes due to its low toxicity and good cathodic potential range.

One of the most customary issues of voltammetric techniques is their susceptibility to disturbances related to the presence of organic substances in environmental samples. The underlying problem is related to the adsorption of organic substances on the sensor surface, which makes it impossible to carry out the measurement properly. Forasmuch as organic matter is an inseparable part of environmental samples, it seems that there is a reasonable need to develop a procedure making it possible to determine Se(IV) in environmental samples abundant in organics without any interference. Until now, only very few articles dedicated to the electrochemical study of selenium which take into consideration the problem linked to the presence of organic substances in the sample have been found in the literature [11,12,13,14,15,18,20,22,30]. In the above-mentioned works, most researchers propose to remove organics prior to the voltammetric detection of Se either by destruction (ultraviolet irradiation, heating with mineral acids) or by separation of humic substances from Se species using chromatographic methods. Nevertheless, both of them take a long time. On the other hand, in the paper [22], interferences from organic matter were efficiently removed by inserting Amberlite XAD-7 resin straight into the voltammetric cell, which curtailed the overall time of measurements. The shortcoming of this procedure was the use of the toxic hanging mercury drop electrode as a working electrode, which considerably restricts its usefulness for measurements. Given the passable results submitted in that paper, in our work, the adsorptive nature of Amberlite XAD-7 resin was likewise used to remove interference generated by humic substances.

Thus, the objective of the present study was to elaborate, optimize, and validate an electroanalytical ASV method based on the differential pulse voltammetry technique for the direct assessment of selenium in natural water samples using an eco-friendly glassy carbon electrode with embedded bismuth film. Modifications based on bismuth films provide a viable alternative for gaining better sensitivity and replacing mercury films, with simpler preparation and operation. In addition to the basic electrolyte, Amberlite resin was added to the voltammetric cell with the analyzed sample, which makes it easy to analyze environmental samples abundant in humic substances as the main component of the organic matrix of natural water samples. In this work, after optimization of all the experimental conditions of the selenium detection procedure and examination of its analytical characteristics, the interferences of humic substances were thoroughly studied and, as proven, efficiently diminished by mixing with resin. Finally, BiFE was applied to the determination of the amount of selenium in certified reference materials and various natural water samples.

## 2. Materials and Methods

### 2.1. Instrumentation

Voltammograms were collected using a µAutolab analyzer (Eco Chemie, Utrecht, The Netherlands), in combination with General Purpose Electrochemical System (GPES) software, operating with a three-electrode cell with a glassy carbon electrode (GCE) (Ø = 1 mm) as substrate for the bismuth film working electrode. A platinum wire was used as a counter electrode. All potentials were quoted against the Ag/AgCl electrode filled with saturated NaCl. The experiments were carried out in a 10 mL electrochemical glass cell, while a magnetic bar rotated at approximately 1000 rpm provided stirring.

Before each series, the GC electrode was polished regularly with abrasive paper of 2500 grit, whereupon using 0.3 μm alumina slurry on a Buehler polishing pad, followed by thorough washing with ultrapurified water. After rinsing, the electrode was sonicated in water for 30 s using an ultrasonic cleaner (Sonic-3, Polsonic, Warszawa, Poland). After drying, the GC electrode was further used to quantify different concentrations of Se(IV). During measurements, the carbon electrode was plated with bismuth film in situ. An Elmetron pH meter CI-316 was used to measure the solution pH values. The cooling thermostat LAUDA ECO SILVER RE 415 S was used for thermostating the tested samples.

### 2.2. Reagents and Solutions

All solutions were freshly prepared by adjusting the concentration to a proper level through dilution using ultrapurified water (resistivity was 18 MΩ cm) supplied by the Milli-Q water ultrapurification system (Millipore, West Lothian, UK). All reagents for the solutions were of analytical reagent (AR) grade. A standard stock solution of Se(IV) at a concentration of 1 g L^−1^ was obtained from Sigma Aldrich. Working solutions of Se(IV) were daily prepared by the appropriate dilution of the stock solution with 1 × 10^−2^ mol L^−1^ of HNO_3_, as required. First, 1 mol L^−1^ CH_3_COOH was prepared from TraceSELECT for trace analysis acetic acid obtained from Sigma Aldrich. A standard solution of 1 g L^−1^ Bi(III) was purchased from Fluka (Buchs, Switzerland). Then, 1 mol L^−1^ NaOH used in pH optimization studies was prepared by dilution of 30% reagent obtained from Merck. The interference effect was checked using standard solutions of 1 g L^−1^ Cd(II), Co(II), Cr(VI), Cu(II), Fe(III), Ga(III), Ge(IV), Hg(II), Mn(II), Mo(VI), Ni(II), Pb(II), Pt(IV), Sb(III), Sn(IV), V(V), Zn(II) (Merck). The influence of organic substances was investigated based on the following humic substances: humic acid sodium salt (HA) obtained from Aldrich as well as natural organic matter (NOM) and river fulvic acid (FA), which were both obtained from the Suwannee River and purchased from the International Humic Substances Society. Amberlite XAD-7 resin obtained from Sigma (St. Luis, MO, USA) was washed four times with ultrapure water before use and dried up at a temperature of 50 °C.

In order to verify the accuracy of the methodology, the following certified reference materials were chosen: TM-25.5 (Lake Ontario water, Environment and Climate Change, Canada), and SPS-SW1 (surface water, Spectrapur Standards As, Oslo, Norway). All water samples were inserted to the supporting electrolyte and analyzed after 120 s shaking with 0.1 g Amberlite XAD-7 resin.

### 2.3. Voltammetric Procedure

For the efficacious removal of organic interferents, a given volume of an analyzed real sample or a synthetic sample (containing Se(IV) and optionally organic interferents), 1 mL of 1 mol L^−1^ acetic acid, 40 µL of 1 g L^−1^ Bi(III), and a suitable volume of ultrapurified water (so that the final volume of the solution was 10 mL) were added to the voltammetric cell, and then, 0.1 g of XAD-7 resin was inserted. After stirring the prepared solution for 120 s, the following sequence of potentials was applied to the electrode: −1.6 V for 5 s and −0.4 V for 60 s. This stage was executed for simultaneously plating bismuth film onto the GC surface and selenium(IV) deposition. Over this time, the solution was stirred using a magnetic stirring bar. Following the preconcentration, the stirrer was switched off, and the solution was left to settle for an equilibrium time of 5 s. After that, the stripping process was carried out by different pulse voltammetry over the potential range from −0.2 to −0.8 V with a step potential of 0.005 V, a modulation amplitude of 0.025 V, and a scan rate of 10 mV/s. After each scan (and before the next measurement), the GC electrode was held at −1.4 V for 10 s and then at +0.3 V for 10 s to allow the reduction and removal of the reduced species from the surface. All measurements were carried out without deletion of oxygen from the solution.

## 3. Results and Discussion

### 3.1. Influence of pH and Supporting Electrolyte Concentration

The nature and type of electrolytes used have a central role in the voltammetric signal of the sensor toward selenium. Hence, in the first stage of optimization of the selenium determination method, the effect of the supporting electrolyte type on the analytical signal of selenium was studied. The influence of electrolyte composition on the peak current of selenium using the in situ plated BiFE was investigated separately. The currents of the responses obtained with the sensor toward a 5 × 10^−8^ mol L^−1^ selenium solution were determined in the following supporting electrolyte with a concentration of 0.1 mol L^−1^ HNO_3_, H_2_SO_4_, H_3_PO_4_, HCl, CH_3_COOH, and acetate buffer pH from 3.6 to 5. Based on the performed measurements, it was found that the highest peak current of Se(IV) and the lowest background level were obtained in the acetic acid solution. Therefore, in the next stage, the effect of CH_3_COOH concentration on the intensity of the Se(IV) peak current was also determined. For this purpose, a series of solutions of acetic acid in the electrochemical cell were prepared at concentrations of 0.05, 0.07, 0.1, 0.15, 0.2, 0.25, and 0.3 mol L^−1^. The results show that a change of CH_3_COOH concentration does not significantly influence the voltammetric signal of selenium, but for a concentration lower than 0.07 mol L^−1^, the reproducibility of the signal was not satisfactory. Considering this, 0.1 mol L^−1^ CH_3_COOH was used as the supporting electrolyte in the succeeding studies.

### 3.2. Examination of Stability of Se(IV) Signal Value in the Presence of XAD-7 Resin

The idea was to eliminate organic matter from the solution by adsorbing it on the resin, with the Se(IV) ions left quantitatively in the solution. Thus, the possible adsorption of Se(IV) on Amberlite XAD-7 resin was examined. The measurements were executed for the samples containing 5 × 10^−8^ mol L^−1^ Se(IV) after blending them for 120 s with 0.1, 0.2, and 0.5 g Amberlite XAD-7 resin. For each sample, the signal of Se(IV) was registered, and the signals of Se(IV) received in the absence and presence of miscellaneous masses of Amberlite XAD-7 resin were collated. It was found that the addition of 0.1 g resin to the sample did not affect the Se(IV) peak current, while a larger quantity of resin present in the voltammetric cell made the analytical signal more unstable. Therefore, it was found that under the applied circumstances, in the presence of 0.1 g of resin, Se(IV) ions stayed quantitatively in the solution, which enabled their quantitative measurement.

### 3.3. Examination of the Se(IV) Signal Intensity Depending on the Temperature

The studies of the selenium signal intensity depending on the temperature were conducted in 0.1 mol L^−1^ acetic acid for a selenium concentration of 3 × 10^−7^ mol L^−1^. The concentration of bismuth was 1.9 × 10^−5^ mol L^−1^. The measurements of selenium signal intensity were performed at different temperatures. All measurements were carried out in a 10 mL vessel thermostated with a cooling thermostat. The temperature was changed in the range of 5–35 °C by every 5 °C. At each tested temperature, the selenium signal was recorded three times. It turned out that in the range of 15 to 35 °C, the selenium signal value is unchanged. However, at a temperature below 15 °C, the selenium signal decreases, and finally, at a temperature equal to 5, it reaches a value of only 30% in relation to the signal obtained at a temperature from the range of 15 to 35 °C. Taking into account the effect of temperature on the efficiency of the accumulation stage, further studies were carried out at room temperature of 20 ± 1 °C.

### 3.4. Choice of Conditions Affecting the Morphology of the Bismuth Film Formed on the Glassy Carbon

BiFEs were introduced to electrochemical research in 2000 as an alternative electrode material replacing more toxic mercury electrodes [45]. The developed voltammetric procedures with the use of the above electrodes have been described in the literature continuously since their first application, which is closely related to the excellent analytical properties of these electrodes [45,46,47,48,49,50,51,52]. They have gained popularity mainly due to their lower toxicity and lower background current in the presence of oxygen compared to mercury electrodes. Using these electrodes does not require deoxidation of the tested solutions during measurements. It should also be mentioned that the bismuth film-modified electrodes are more stable than mercury electrodes, so they can be used in flow systems with fast mass transport as well as in the field. Furthermore, these electrodes are characterized by the high sensitivity and repeatability of signals, and detection limits gained with their use do not differ from those obtained with mercury electrodes. BiFEs have been obtained by applying a bismuth film on a solid electrode made of glassy carbon [45,46,47,48,49], carbon paste [48,50], graphite pencil [48], impregnated graphite [51], and screen-printed carbon electrode [52]. The bismuth film can be applied by two methods, in situ or ex situ, and glassy carbon electrode is the most often used as a substrate for bismuth film [45,46,47,48,49]. The in situ method relies on introducing soluble salt of bismuth to the tested sample. After that, from the same solution, bismuth film is applied to the electrode, and at the same time, the analyte is accumulated. This way is used when the film formation conditions are close to the optimal voltammetric measurement conditions. The ex situ method is applicable when it is difficult to select conditions that allow to form the bismuth film and accumulate the analyte in a single measurement. In this method, it is necessary to prepare an additional solution from which the bismuth film on the electrode substrate is generated. After washing, the bismuth film electrode is placed in the analyzed solution, and the voltammetric measurement is carried out. The ex situ method is more laborious, extends the total measurement time, and generates the additional consumption of reagents compared to the in situ method. The choice of the appropriate method depends on the pH of the tested solution. The best pH for bismuth film generation is acidic medium. This is due to the fact that Bi(III) salts undergo hydrolysis in an inert and alkaline pH. Thus, when a non-acidic pH is required, the ex situ method of applying the bismuth film is preferred. However, the generation of a bismuth film by the in situ method at non-acidic pH is possible provided that tartrate is added to the analyzed solution. This is due to the fact that Bi(III) forms a stable complex with the tartrate, and thus, it does not undergo hydrolysis.

In this work, the measurements are carried out in an acidic medium (0.1 mol L^−1^ acetic acid pH = 2.8); therefore, the bismuth film is formed in situ on the glassy carbon (GC). The thickness of the bismuth film formed on the GC electrode affects the height and shape of the peaks obtained in the course of determination of metal ions by the anodic voltammetry method. The thickness of the bismuth film formed is greatly influenced by the concentration of the bismuth(III) salt introduced into the analyzed solution as well as deposition potential of bismuth film onto the electrode surface [45]. Therefore, these parameters have been precisely researched and optimized.

#### 3.4.1. Influence of Bismuth Concentration

**Figure 1:**
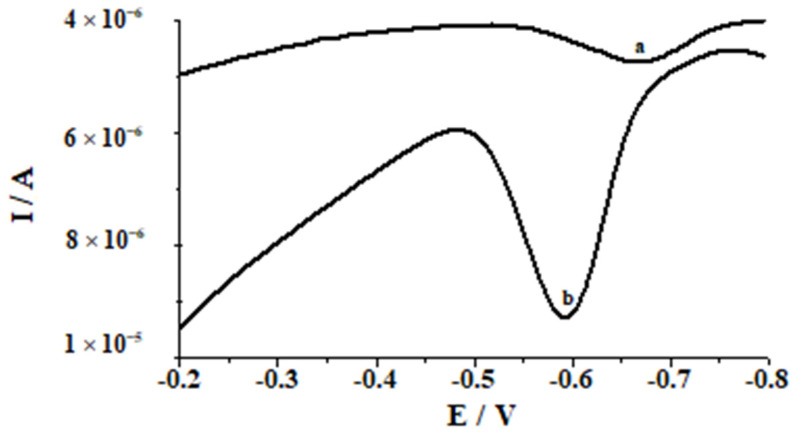
Voltammograms of Se(IV) obtained from solution: (a) without addition of Bi(III); (b) upon addition of 1.9 × 10^−5^ mol L^−1^. Steady concentration: 3 × 10^−7^ mol L^−1^ Se(IV), 0.1 mol L^−1^ acetic acid.

Preliminary studies designed to compare the efficiency of the bare glassy carbon electrode and of the electrode modified with bismuth film were conducted in 0.1 mol L^−1^ acetic acid for a selenium concentration of 3 × 10^−7^ mol L^−1^. To modify the glassy carbon electrode, the in situ approach was chosen because of its fast, easy, and reproductive modification and the fact that the extra step needed for ex situ modification is excluded. The results depicted in Figure 1 showed that bismuth film makes the glassy electrode more sensitive for selenium. Thus, the sensor applicability for selenium determination in natural water samples is improved. The gain on sensitivity when bismuth films are used can be related to the co-accumulation of bismuth with selenium, forming binary alloy [53].

**Figure 2:**
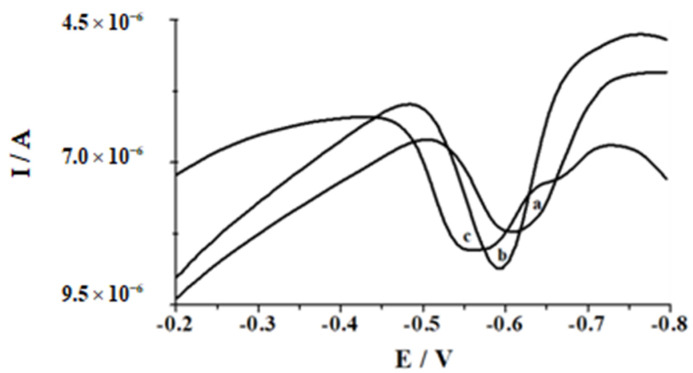
The voltammograms illustrating the influence of Bi(III) concentration on the peak current and potential of 3 × 10^−7^ mol L^−1^ Se(IV). Concentration of Bi(III): (a) 4.8 × 10^−6^ mol L^−1^; (b) 1.9 × 10^−5^ mol L^−1^; (c) 1.9 × 10^−4^ mol L^−1^. Steady concentration: 3 × 10^−7^ mol L^−1^ Se(IV), 0.1 mol L^−1^ acetic acid.

Then, to examine the relationship between bismuth concentration and peak current of 5 × 10^−8^ mol L^−1^ selenium, the quantity of bismuth in the sample was varied in the range concentration from 4.8 × 10^−6^ mol L^−1^ to 2.4 × 10^−4^ mol L^−1^. On examining the above dependence, one can notice that the peak current increased with increasing bismuth concentration up to 1.9 × 10^−5^ mol L^−1^ and finally decreased at higher concentrations. As can be additionally observed in Figure 2, the voltammetric peak potential shifted to more positive potentials with increasing the bismuth concentration. On the basis of these results, the Bi(III) concentration of 1.9 × 10^−5^ mol L^−1^ was chosen as the optimum one for further research.

#### 3.4.2. Conditions of the Accumulation Step

**Figure 3:**
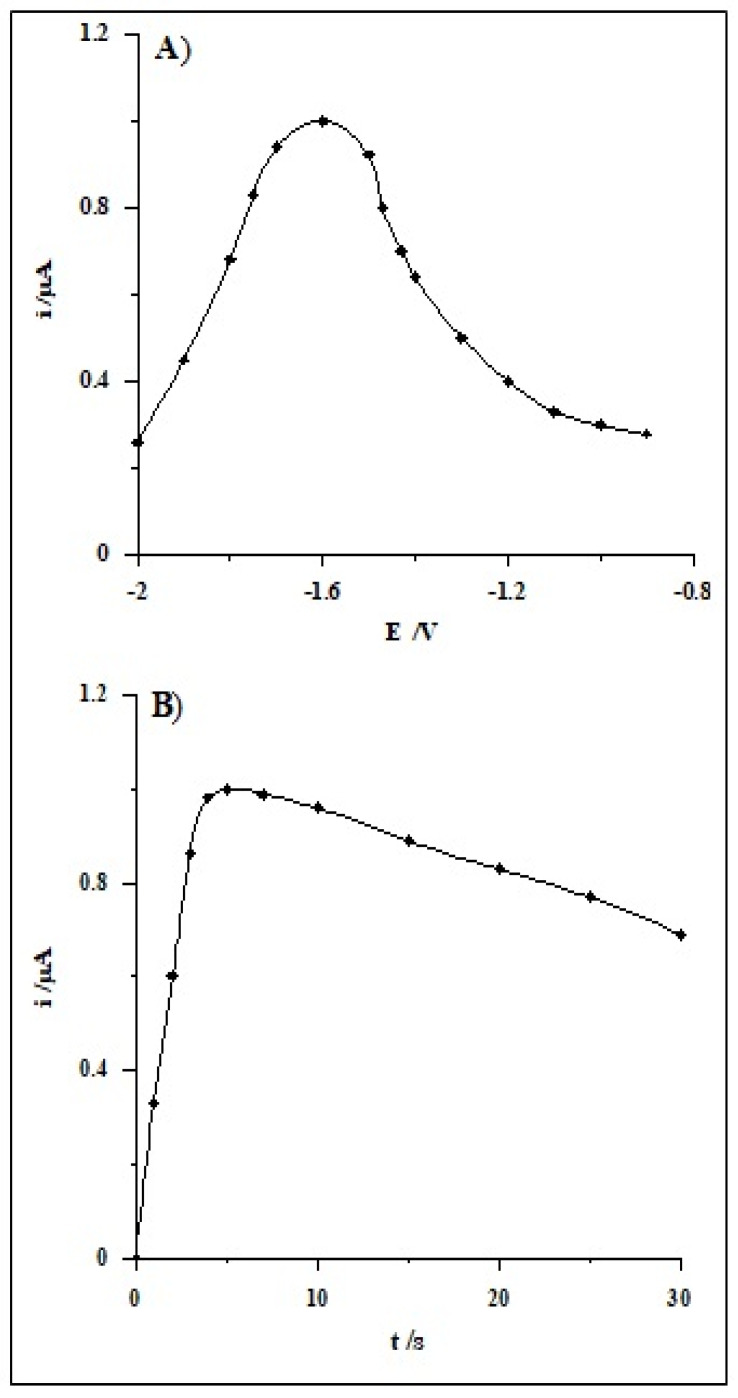
The impact of first accumulation potential (**A**) and its time (**B**) on the voltammetric signal of Se(IV). Steady concentration: 1 × 10^−7^ mol L^−1^ Se(IV), 0.1 mol L^−1^ acetic acid, 1.9 × 10^−5^ mol L^−1^ Bi(III).

The influence of potential and time on Bi film formation and the influence of the accumulation of Se(IV) on selenium peak current was investigated for the Se(IV) concentrations of 1 × 10^−7^ mol L^−1^. Preliminary measurements demonstrated that a higher selenium signal was received when the accumulation step was carried out using two different potentials, wherein the first potential should be more negative than the second one. The first potential ranged from −0.9 to −2.0 V for a steady time of 5 s. The measurements were executed applying the second potential for 60 s at −0.4 V and the solution comprising 0.1 mol L^−1^ acetic acid and 1.9 × 10^−5^ mol L^−1^ Bi(III). As it can be noticed in Figure 3A, the maximum signal of Se(IV) was achieved for the potential of −1.6 V, so for subsequent measurements, this level of potential was adopted. Afterwards, the impact of time of the first potential was checked for the selected potential in the range from 0 to 30 s. The obtained results presented in Figure 3B indicate that the peak current of selenium increases with an extension in the lead film creation time up to 5 s, whereas at longer times, the peak decreases.

**Figure 4:**
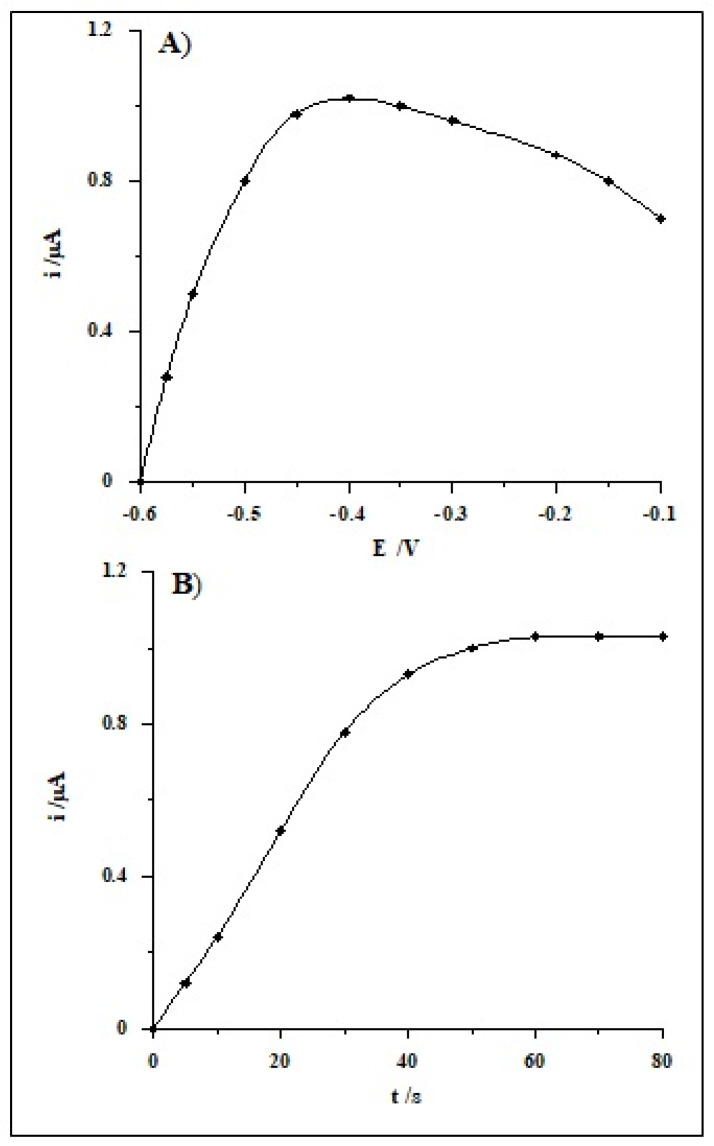
The impact of second accumulation potential (**A**) and its time (**B**) on the voltammetric signal of Se(IV). Steady concentration: 1 × 10^−7^ mol L^−1^ Se(IV), 0.1 mol L^−1^ acetic acid, 1.9 × 10^−5^ mol L^−1^ Bi(III).

Then, the dependence of the selenium peak current on the second accumulation potential was studied changing it from −0.1 to −0.6 V for a steady period of time of 60 s. The highest analytical signal was obtained at the potential of −0.4 V, and it was chosen as the optimum one, while for lower and higher values, the signal little by little decreased (Figure 4A). The time of the second potential, ranging from 0 to 80 s, was subsequently examined. On the basis of the executed measurements, it was found that the Se(IV) analytical signal increased linearly up to 60 s, whereas at longer times, the peak was unaltered (Figure 4B).

### 3.5. Calibration Data

**Figure 5:**
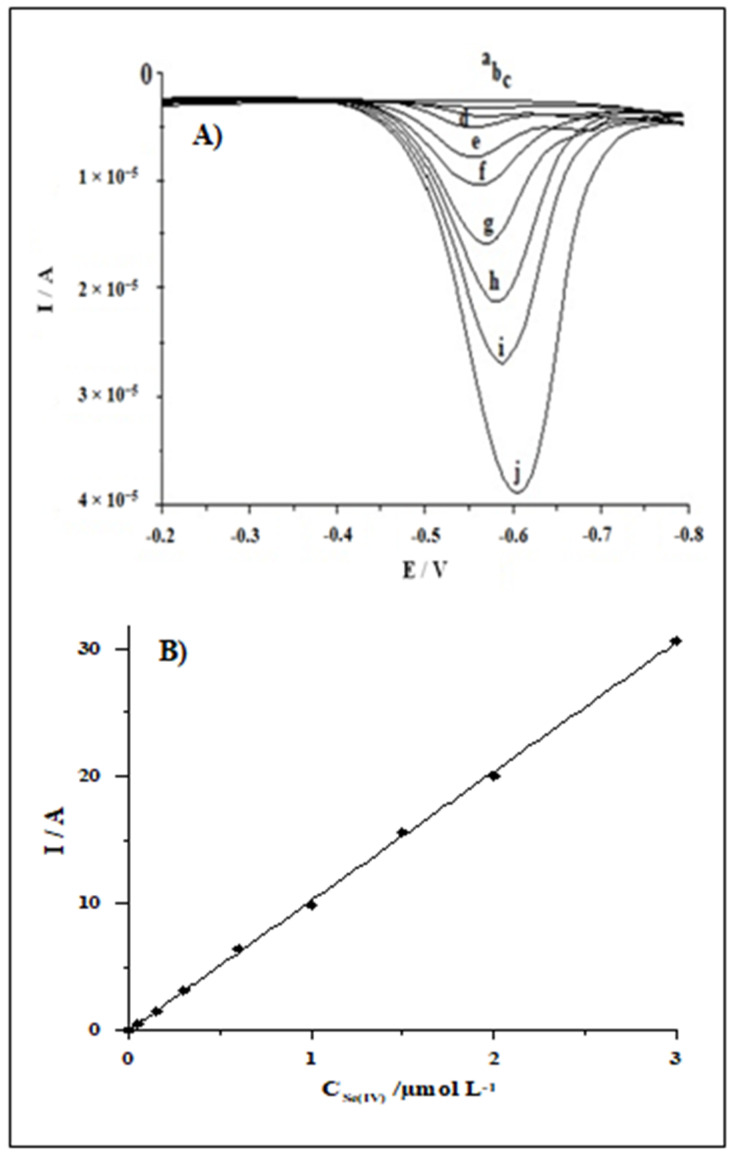
The voltammograms obtained at the BiFE in the supporting electrolyte containing increasing concentrations of Se(IV) (**A**). Concentration of Se(IV): 0 (a), 0.003 (b), 0.05 (c), 0.15 (d), 0.3 (e), 0.6 (f), 1 (g), 1.5 (h), 2 (i), 3 (j) µmol L^−1^. Supporting electrolyte: 0.1 mol L^−1^ acetic acid, 1.9 × 10^−5^ mol L^−1^ Bi(III). Inset the corresponding calibration plots (**B**).

The analytical features of the proposed sensor were evaluated. The calibration plot was recorded under the optimized conditions as described above (0.1 mol L^−1^ acetic acid containing 1.9 × 10^−5^ mol L^−1^ Bi(III) and 0.1 g Amberlite XAD-7 resin, successive accumulation potentials: −1.6 V for 5 s followed by −0.4 V for 60 s). The voltammograms obtained for increasing concentrations of Se(IV) and the respective calibration plot are illustrated in Figure 5. The linearity of the calibration graph was maintained in a wide range, from 3 × 10^−9^ to 3 × 10^−6^ mol L^−1^. The equation of the calibration graph was *y* = 10.15x + 0.04, where *y* and *x* are the peak current (µA) and Se(IV) concentration (µmol L^−1^), respectively. Each point of the calibration graph is the average of three replications. The linearity of the calibration curve is confirmed by the correlation coefficient (*r* = 0.9995). The relative standard deviation (RSD) from five determinations of selenium at a concentration of 5 × 10^−8^ mol L^−1^ was 4.2%. The detection limit estimated from three times the standard deviation at a low Se(IV) concentration was about 8 × 10^−10^ mol L^−1^. For short periods (one day), the stability of the sensor is splendid. Over longer periods (five consecutive days), for about 5 h of measurements per day, the signal sensitivity fluctuates by about 13%.

The analytical parameters of the proposed procedure and of other stripping voltammetric procedures using mercury free working electrodes for trace selenium ions determination are compared in Table 1.

### 3.6. Selectivity

Analytical selectivity is one of the important parameters that affect the accuracy of the methodology. Therefore, the selectivity of the bismuth modified glassy carbon electrode for the determination of selenium was evaluated using concomitant metal ions. The impact of co-existing ions in the sample solution was investigated using a constant concentration of 2 × 10^−7^ mol L^−1^ Se(IV) and a different amount of various common ions. The tolerance level was set as the greatest concentrations that result in an error of ±5% in the determination of Se(IV). The recoveries of Se(IV) were almost quantitative in the presence of many tested ions as indicated in Table 2. Only Cd(II), Co(II), Cu(II), Pb(II), and Zn(II) showed significant interference in the sensor response. To avoid interference from these cations, 2 × 10^−4^ mol L^−1^ EDTA was additionally added to the solution. It was found that after the addition of EDTA, the response of selenium remains the same, and most importantly, interference from Cd(II), Co(II), Pb(II), and Zn(II) was completely removed. Merely the interference caused by Cu(II) has not been completely removed but, the addition of EDTA to the analyzed sample made it possible to determine selenium in the presence of 2 × 10^−7^ mol L^−1^ Cu(II).

### 3.7. Influence of Organic Compounds

The interference generated by organic compounds during Se(IV) voltammetric determination was closely investigated. Out of the organic compounds present in environmental samples, humic substances are favorable. Humic substances belong to heterogeneous organic substances with high molecular weight, which are the most extensively distributed on the earth. These substances are generally strongly adsorbed on the electrode surface in voltammetric procedures, fouling it, and as a result, they impact the voltammetric signal obtained in speciation investigations of metals in natural samples [46]. Thus, commercially available humic substances such as humic acids (HA), fulvic acids (FA), and natural organic matter (NOM) were chosen to study their effect on the peak current of selenium. The measurements were carried out under standard circumstances for samples containing a fixed concentration of selenium equal to 5 × 10^−7^ mol L^−1^ Se(IV) and changeable concentrations of FA, HA, and NOM in the absence or presence of 0.1 g Amberlite XAD-7. The obtained results are summarized in Table 3.

By analyzing the data collected in the table, we can conclude that in the presence of Amberlite XAD-7 resin, the concentrations of all humic substances tested, even as high as 10 mg L^−1^, do not disturb the analytical signal, whereas in the absence of resin, this signal decreases up to about four, three, and two times in the case of humic acids, fulvic acids, and organic matter, accordingly.

### 3.8. Analytical Applications

**Figure 6:**
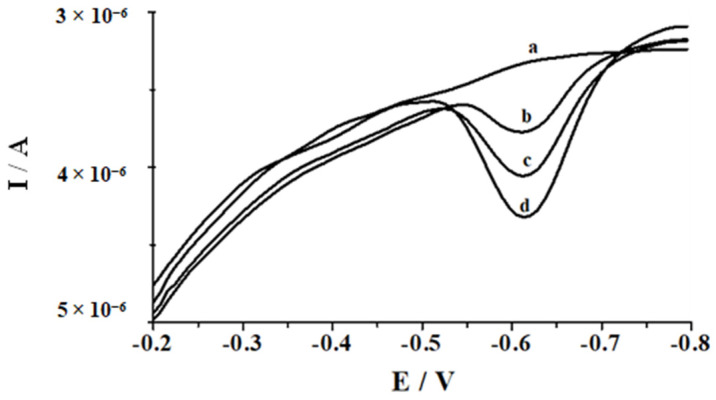
The voltammograms obtained in the course of selenium determination in certified water reference material TM-25.5: (a) blank (0.1 mol L^−1^ acetic acid, 1.9 × 10^−5^ mol L^−1^ Bi(III)); (b) as (a) + TM-25.5 (ten-fold dilution); (c) as (b) + 3.5 × 10^−8^ mol L^−1^ Se(IV); (d) as (b) + 7 × 10^−8^ mol L^−1^ Se(IV). Potential and time of deposition: −1.6 V for 5 s followed by 0.4 V for 60 s.

Under the optimized experimental conditions, the method was applied for the determination of Se(IV) in two types of the certified reference materials: SPS SW-1 (surface water) and TM-25.5 (lake water), with different concentrations of Se(IV) using the standard addition technique. The certified reference materials were added straight to the voltammetric cell without any pretreatment and diluted with supporting electrolyte. Since solutions of both certified reference material comprise nitric acid after the addition of CRM to the supporting electrolyte, its pH was adjusted to the value of 2.8 using 1 mol L^−1^ NaOH. Under such conditions, the voltammogram was recorded, showing the selenium peak corresponding to its concentration in the solution. A comparison of the obtained results with the certified values in Table 4 is presented. The collected data show that the measured values are in good agreement with the certified concentrations. Examples of the voltammograms registered in the course of Se(IV) determination in TM-25.5 CMR are shown in Figure 6.

Additionally, natural water samples such as Bystrzyca river water, tap water, mineral water, and rain water, all spiked with selenium(IV), were analyzed according to the described procedure. The results from Se(IV) determination are listed in Table 5. The recovery of Se(IV) ranged from 100 to 109%. All obtained results confirm the correctness of the presented procedure and the analytical usefulness in real water sample analysis for Se(IV) determination.

## 4. Conclusions

This work presents a low-cost alternative to more expensive spectrometric methods for the determination of selenium in natural water samples. The developed ASV method is rapid, sensitive, and allows for direct Se(IV) quantification in natural water samples without any sample pretreatment, which was confirmed by the passable results obtained for the analysis of certified reference materials, such as surface water and lake water, and fresh natural water samples, such as river water, tap water, mineral water, and rain water. The use of the in situ plated bismuth film electrode instead of mercury-based electrodes is an attempt to perform eco-friendly electrochemical determination of selenium. The BiFE exhibits a linearity response over a relative wide selenium concentration range (3 × 10^−9^–3 × 10^−6^ mol L^−1^) and shows a detection limit of 8 × 10^−10^ mol L^−1^. Amberlite XAD-7 resin allows solving problems such as the adsorption of organics on the electrode surface, and therefore, the proposed method provides an improvement in the existing anodic stripping techniques for selenium determination. Moreover, the described procedure is characterized by good selectivity, which was found as a result of the analysis of the influence of multiple foreign ions. Meanwhile, the lack of the need to deoxygenate the solution allows it to be adapted to field analyses.

## Figures and Tables

**Table 1:** Comparison of parameters of the proposed procedure with other stripping voltammetric procedures using ecofriendly mercury free working electrode for trace Se(IV) determination. The procedures are arranged according to the growing detection limit.

Working Electrode	Voltammetric Method	Detection Limit [nmol L^−1^]	Linear Range [nmol L^−1^]	Accumulation Time [s]	Analytical Application	Ref.
CCE-DAN	AdSV	0.25	1.27–250	90	natural and mineral water	[52]
GEMO-PN	ASV	0.60	50–1000	200	pharmaceuticals	[44]
BiFE	AdSV	0.63	25–633	90	-	[47]
BiFE	ASV	0.80	3–3000	65	SPS-SW1 and TM-25.5 certified reference materials, river, tap, mineral, and rain water	This work
AuµE and AuE	ASV	0.85	5–100	300	SPS-SW1 certified reference material	[37]
AuNDs/P-rGO	ASV	0.90	3–300	-	seawater and standard artificial seawater	[43]
BiFE	AdSV	1.3	25–380	300	-	[48]
GCE- AuNPs	ASV	1.5	126–633	120	seawater	[41]
RAgABE	CSV	1.9	12.7–127	300	SPS-SW1 and SPS-SW2 certified reference materials	[28]
AgE	CSV	2.5	63–506	1800	-	[27]
Au UME	ASV	5.3	0–1266.5	-	-	[40]
AuµE	ASV	6.3	0–190	20	-	[38]
GCE-AuNPs	ASV	8.1	-	60	real water samples	[42]
AuE	ASV	9.9	-	10	supplements	[33]
GCE-rGO	ASV	10	-	240	water samples	[46]
AuE	ASV	60	200–1000	300	river water	[36]
SPGE	ASV	62	127–3165	300	tap water	[45]
AuE	ASV	-	6.3–3683.5	180	-	[34]
CuµE	CSV	-	5000–50,000	15	-	[29]

**Table 2:** The effect of interfering ions on DPV response of selenium at BiFE. Recovery as a ratio of selenium signal in the presence of interfering ions to the signal obtained in the absence of these ions upon rounding to the whole number (expressed as a percentage).

Interfering Ions	Recovery of DPV Response of Selenium (%)		
	Interferent/Selenium Concentration		
	1:1	5:1	10:1
Cd(II)	73	39	27
Cd(II) *	100	100	100
Co(II)	78	48	34
Co(II) *	100	100	100
Cr(VI)	102	101	102
Cu(II)	17	10	lack of signal
Cu(II) *	100	44	10
Fe(III)	98	68	53
Ga(III)	100	82	78
Ge(IV)	102	101	100
Hg(II)	100	100	95
Mn(II)	100	100	98
Mo(VI)	101	69	52
Ni(II)	100	98	96
Pb(II)	67	38	22
Pb(II) *	100	100	100
Pt(IV)	103	104	104
Sb(III)	100	100	100
Sn(II)	100	100	99
V(V)	100	100	99
Zn(II)	75	54	38
Zn(II) *	100	100	100

* the interfering ion effect tested in the presence of 2 × 10^−4^ mol L^−1^ EDTA.

**Table 3:** Relative signal of Se(IV) in the presence of FA, HA, and NOM in relation to the signal obtained in the absence of these substances. Experiments were performed in the presence and absence of Amberlite XAD-7 resin for a selenium concentration of 5 × 10^−7^ mol L^−1^.

Humic Substances Added		Relative Signal of Se(IV) ± RSD [%]	
Type of Substances	Concentration [mg L^−1^]	In the Absence of Resin	In the Presence of Resin
HA	0.5	98.0 ± 3.3	99.9 ± 2.9
	1	74.3 ± 5.0	97.7 ± 4.1
	2	50.9 ± 2.3	98.6 ± 4.5
	3	39.2 ± 3.8	108.5 ± 4.6
	5	35.0 ± 5.4	100.0 ± 4.0
	10	24.0 ± 4.3	98.7 ± 3.8
FA	0.5	94.1 ± 3.9	100.9 ± 1.7
	1	74.7 ± 5.1	108.3 ± 4.6
	2	66.6 ± 4.5	104.7 ± 3.9
	3	60.5 ± 3.6	106.0 ± 4.8
	5	52.0 ± 4.2	105.3 ± 3.6
	10	33.1 ± 4.5	99.6 ± 4.6
NOM	0.5	88.9 ± 3.7	102.4 ± 2.5
	1	79.7 ± 4.5	101.2 ± 4.7
	2	68.6 ± 5.3	104.7 ± 3.9
	3	58.8 ± 5.1	102.0 ± 5.3
	5	54.4 ± 3.8	97.8 ± 3.8
	10	49.5 ± 5.0	104.5 ± 5.6

**Table 4:** Results obtained during the determination of Se(IV) in certified reference materials.

Certified Reference Material	Measured Value ± SD (*n* = 3) [µg L^−1^]	Certified Value ± SD (*n* = 3) [µg L^−1^]
SPS SW-1	1.95 ± 0.11	2.00 ± 0.02
TM-25.5	30.0 ± 4.1	29.2 ± 3.4

**Table 5:** Analytical results of Se(IV) determination in fresh natural water samples by the proposed procedure in the presence of 0.1 g Ambertite XAD-7 resin. The samples were analyzed after tenfold dilutions using standard addition methods.

Sample	Concentration of Added Se(IV) [nmol L^−1^]	Concentration of Found Se(IV) [nmol L^−1^]	Recovery [%]	RSD (*n* = 5) [%]
Bystrzyca riverwater	50	54	108	6.1
	100	109	109	5.2
Tapwater	50	51	102	5.8
	100	105	105	6.0
Mineralwater	50	50	100	3.3
	100	107	107	4.2
Rainwater	50	51	102	3.5
	100	102	102	4.9

## Sample Availability

Samples of the TM-25.5 and SPS-SW1 are available from the authors.
